# Evaluation of bone health in terms of osteoporosis in adult patients with neuromuscular disease

**DOI:** 10.55730/1300-0144.5794

**Published:** 2023-11-11

**Authors:** Özgür Zeliha KARAAHMET, Eda GÜRÇAY, Yasemin TOMBAK YILDIZKAN, Damla CANKURTARAN, Zeynep Tuba BAHTİYARCA, Ebru UMAY

**Affiliations:** 1 Department of Physical Medicine and Rehabilitation, University of Health Sciences, Etlik City Hospital, Ankara, Turkiye; 2 Department of Physical Medicine and Rehabilitation, University of Health Sciences, Gaziler Training and Research Hospital, Ankara, Turkiye

**Keywords:** Bone health, myopathy, neuronopathy, neuropathy

## Abstract

**Background/aim:**

There are no current guidelines to help clinicians decide whether patients with adult neuromuscular disease (NMD) should be screened or treated for osteoporosis (OP). This study was undertaken to investigate the presence of OP in patients with various types of NMD and to examine the relationship between OP evaluation parameters and functional status, daily living activities, balance, and ambulation levels.

**Materials and methods:**

This cross-sectional study included 45 patients with NMDs. The patients were divided into 3 groups, depending on the affected component of the motor unit (neuronopathy group, neuropathy group, and myopathy group). The laboratory and demographic data were recorded from patient files. Functional level, pain, muscular strength, balance, and daily living activity scores were evaluated. The presence of OP was quantified using bone densitometry, fracture history, and biochemical parameters. Clinical findings were correlated with laboratory and dual-energy X-ray absorptiometry (DEXA) findings.

**Results:**

The mean hip T-score was −1.20, and the mean lumbar spine (L1–L4) T-score was −0.95 in all groups. Six patients with T-score values of −2.5 or below were detected. Vitamin D level was found to be low in all patient groups, especially in the myopathy group, but there was no significant difference (p > 0.05). There was a negative correlation between hip T-score and the frequency of falling (r = −0.604, p = 0.022), while a positive correlation was found between hip T-score and the age at which independent walking was no longer possible (r = 0.900, p = 0.037).

**Conclusion:**

OP is often overlooked in NMD patients with neurological problems and a high risk of falling. These patients should be screened for bone health and fragility.

## 1. Introduction

Neuromuscular diseases (NMDs) are complex, heterogeneous, acquired, or inherited disorders that affect the motor unit. NMD is an umbrella concept that includes different divisions of disorders influencing anterior horn cells, peripheral nerves, neuromuscular junctions, and skeletal muscles. With a prevalence of less than 1 in 2000, NMDs are rare diseases; however, there is a need to challenge the existing diagnostic and therapeutic approaches to address several clinical and functional issues [1].

Osteoporosis (OP) is a disease defined by decreased bone mass and changes in the microarchitecture of bone tissue that cause bone fragility and an increased risk of fractures [2]. Today, OP is a common health matter in industrialized countries, affecting approximately 30% of women and 8% of men over the age of 50 in Europe [3]. Poor bone health is often a significant problem for patients with NMD [4]. It has been reported that bone mineral density decreases and fracture risk increases in amyotrophic lateral sclerosis (ALS), Duchenne muscular dystrophy (DMD), and spinal muscular atrophy (SMA) [4–6].

A diagnosis of OP is difficult before a fragility fracture occurs [7]. The majority of patients admitted to the hospital with fragility fractures do not know that they have low bone mineral density [8]. OP may induce severe morbidity and mortality if not properly diagnosed and treated. The available evidence is discussed with a focus on abnormal calcium metabolism, increased fracture risk, and the prevalence of both scoliosis and hypovitaminosis D in ALS, DMD, and SMA. Currently, there are no guidelines to help clinicians decide whether these patients require screening or intervention for bone density reduction. Existing studies on fracture risk in patients with NMDs are limited to patients with DMD with glucocorticoid exposure and patients with ALS [9]. Although clinical guidelines exist for the screening and treatment of OP for a variety of neurological diseases, NMDs have not yet been included as supporting data are scarce. Therefore, the purpose of this study is to investigate in which NMD group the presence of OP is more common. Furthermore, it aims to examine the relationship of OP to daily living activities, ambulation levels, and balance.

## 2. Materials and methods

### 2.1. Study design

This single-center, cross-sectional study included 45 patients with NMDs who were admitted to the NMD clinic between November 2020 and December 2021. The laboratory and demographic data of the patients were analyzed from the patient files. Clinical evaluations were made when the patient applied to the clinic. The study was conducted within the scope of the Declaration of Helsinki. Diskapi Yıldırım Beyazıt Training and Research Hospital ethics committee approval and informed consent forms were obtained for the study.

### 2.2. Participants

All of the hospitalized NMD patients were postmenopausal women over 37 years old and men over 50 years old. Those with motor neuron involvement, peripheral neuropathy, and myopathy were included. This study excluded patients with neuromuscular junction involvement or incomplete information. Also, patients who were previously diagnosed with OP and patients who had used steroid therapy for a long time were not included in the study.

The patients were divided into 3 groups according to the motor unit affected component. Group 1 (neuronopathy group, n = 11) had motor neuron involvement, group 2 (neuropathy group, n = 13) had peripheral neuropathies, and group 3 (myopathy group, n = 21) had myopathies.

### 2.3. Evaluation parameters

Demographic and clinical characteristics of the patients including the following variables were recorded from the patient files: age, sex, age at diagnosis, body mass index (BMI), presence of comorbidity (diabetes mellitus, hypertension, hyperlipidemia, or hypothyroidism), age at which independent walking ceased, number of falls in the previous six months, and any history of bone fracture.

Pain level was assessed using the visual analog scale (VAS) [10], and muscle strength was evaluated according to the Medical Research Council (MRC) test [11]. The Brooke scale was used to grade upper extremity function [12], and the Vignos scale was used to measure lower extremity function [13]. The functional independence measure (FIM) was used to determine degree of daily life activity [14], the Berg balance scale (BBS) helped to measure balance and fall risks [15], and the functional ambulation scale (FAS) was used for ambulation level assessment [16].

### 2.4. Laboratory parameters

In terms of the laboratory parameters for each participant, the following ranges were noted as normal: calcium, 8.6–10.2 mg/dL; alkaline phosphatase (ALP), 35–104 U/L; 25-OH vitamin D3, 30–80 μg/L; parathyroid hormone (PTH), 12–88 ng/L; aspartate aminotransferase (AST), 0–32 U/L; and alanine aminotransferase (ALT), 0–33 U/L. These normal ranges were determined by the Diskapi Yıldırım Beyazıt Training and Research Hospital Biochemistry Laboratory [17].

Bone mineral density (BMD) results obtained from the lumbar vertebrae (in the anterior position between L1 and L4) and the proximal femur with a dual-energy X-ray absorptiometry (DEXA) device (Hologic) were evaluated as g/cm^2^ and T scores were determined according to the peak young adult bone density value.

### 2.5. Data analysis

The data were analyzed using the SPSS 22.0 for Windows (IBM Corporation, Chicago, IL, USA). The conformity of continuous variables to a normal distribution was examined using the Shapiro–Wilk test. Descriptive statistics were shown as mean ± standard deviation (SD) or median (minimum–maximum) for continuous variables, and as frequency (n) and percentage (%) for nominal and categorical variables. An independent simple t-test was used for normally distributed continuous variables and the Mann–Whitney U test was used for nonnormally distributed variables. Comparisons for nominal and categorical variables were evaluated with χ^2^ and Fisher’s exact tests. The relationship between vitamin D, PTH, and DEXA measurements and evaluation parameters was evaluated with Pearson’s (BMI) and Spearman correlation tests. with A value of p < 0.05 was considered statistically significant.

## 3. Results

The demographic and clinical characteristics of the patients are shown in Tables 1 and 2, and there were no significant differences between the groups (p > 0.05). The laboratory and DEXA parameters of the patients for OP evaluation are shown in Table 3. It was determined that vitamin D levels were low in all patient groups. Although it was not statistically significant, this decrease was most pronounced in the myopathy group. Additionally, the myopathy group showed the most significant PTH elevation compared to the other groups (p < 0.05), and the AST levels were higher in the myopathy group (p < 0.05).

The mean hip T-score was −1.20, and the mean lumbar spine (L1–L4) T-score was −0.95 in all groups. Six patients with T-score values of –2.5 and below were detected. The correlation results of the clinical and laboratory findings are noted in Table 4. A positive correlation was found between hip T-score and the age at which independent walking ceased (r = 0.900, p = 0.037), and a negative correlation between hip T-score and the number of falls (r = −0.604, p = 0.022). Although p = 0.05, the correlation between the BBS and the total hip T-score is very close to statistical significance (r = 0.949, p = 0.05).

## 4. Discussion

In the current study, we evaluated the presence of OP in patients with NMD, and in which NMD group it is more common; also the relationship between OP and the patients’ activities of daily life, ambulation level, and balance was examined. No difference was found between the groups in terms of OP. While no correlation was found between OP parameters and FIM and FAS values, a positive correlation was found between balance and hip T-score.

We have not encountered any similar study in the literature to directly compare our results. The relationship between childhood NMD and OP has previously been investigated, especially with DMD. In one study, low bone density and fractures were identified as important problems in children with NMD, and the mentioned risk factors included weakness, decreased mobility, glucocorticoid use, and vitamin D deficiency [18]. In other studies that investigated the relationship between adult NMD and OP, comparisons were made between healthy controls and a specific disease, such as ALS or chronic inflammatory demyelinating polyradiculoneuropathy (CIDP). Caplliure-Llopis et al. found that all bone parameters evaluated in ALS patients were statistically significantly lower than those of healthy controls [19]. Kim et al. found that BMD was significantly lower in CIDP patients than in normal controls. [20].

In our study, no correlation was found between OP parameters and ambulation level or daily living activities. Similarly, Aparicio et al. studied boys with DMD who were ambulatory, did not use assistive devices, and had never used steroids. The study showed that 80% had decreased bone density of the proximal femur and 50% had decreased bone density of the spine, compared with the normative data for the age group [21]. Lee et al. found that the skeletal complications of NMD lead directly and systemically to declined function rather than being local or indirect [22]. Thus, a similar mechanism presenting to the pathogenesis of both neurodegeneration and OP may be suspected. The hypothesis that neurodegenerative disorders are associated with OP is controversial [23]. However, our study showed a positive correlation between hip T-score and both the age at which independent walking ceased and the BBS; it also showed a negative correlation between hip T-score and number of falls. We observed that a negative development in these parameters reduces the T-score. Although it is thought to be systemic rather than reduced in functionality, this issue remains unclear.

We further found that vitamin D levels were low in all patient groups, especially in the myopathy group. Myopathy related to severely low serum vitamin D levels is well-documented in the literature. While vitamin D deficiency itself causes myopathy, vitamin D deficiency may increase the severity of the disease in myopathy patients. Besides the effect of vitamin D on bone and mineral metabolism, it also has antiproliferative, immunomodulatory, and antiinflammatory functions by binding to the vitamin D receptor. These roles may also have a protective effect in myopathies [24–25]. In patients with NMD, vitamin D levels should be checked and vitamin D replacement therapy should be provided for those with low levels.

Although our study has shortcomings in that it is a single-center study with a low number of patients, we believe that the research is still valuable because of the rarity of these diseases. There is only a small number of studies evaluating OP in adult NMD, and this is the first to compare all the NMD groups.

Unfortunately, it is possible that OP issues will be ignored when treating those with NMD. If this results in a bone fracture, the patient may experience a vicious cycle of limitation in ambulation and an increase in OP. Therefore, when evaluating NMD, it is necessary to screen for osteoporosis at the first examination; this simple step can possibly help maintain a good quality of life for the NMD patients for a longer time.

## Figures and Tables

**Table 1:** Demographic characteristics of the patients.

Variables	Total (n = 45)	NNP (n = 11)	NP (n = 13)	MP (n = 21)	NNP–NP p	NNP–MP p	NP–MP p

Age median (min–max)	50 (37.0–72.0)	56 (37.0–72.0)	52 (38.0–67.0)	50 (37.0–69.0)	0.871	0.195	0.132

Sex n (%)							
Female	26 (57.8)	6 (54.5)	7 (54.0)	12 (57.0)	0.974	0.557	0.614
Male	19 (42.2)	5 (45.5)	6 (46.0)	9 (43.0)			

Occupation n (%)							
White-collar	14 (31.1)	3 (27.2)	3 (23.1)	8 (38.1)	0.473	0.276	0.255
Blue-collar	6 (13.3)	4 (36.4)	2 (15.3)	0			
Housewife	16 (35.6)	2 (18.2)	4 (30.8)	10 (47.6)			
Retired	9 (20.0)	2 (18.2)	4 (30.8)	3 (14.3)			

Age at diagnosis median (min–max)	27.0 (1.5–68.0)	37.16 (1.5–68.0)	33.0 (8.0–52.0)	24.45.0 (3.0–52.0)	0.722	0.114	0.173

BMI mean ± SD	24.41 ± 5.38	23.66 ± 3.59	23.41 ± 6.68	23.89 ± 5.24	0.995	0.447	0.628

Presence of comorbidity, n (%)	23 (51.1)	5 (45.5)	4 (30.8)	14 (66.7)	0.698	0.865	0.156

Presence of OP n (%)	6 (13.3)	1 (9.1)	2 (18.2)	3 (14.3)	0.554	0.870	0.802

Falls n (%)	21 (46.7)	5 (45.5)	5 (38.5)	11 (52.4)	0.674	0.881	0.723

Bone fracture n (%)	8 (17.8)	3 (27.3)	3 (23.1)	2 (9.5)	0.897	0.241	0.272

SD = standard deviation; min = minimum; max = maximum; n = number; NNP = neuronopathy; NP = neuropathy; MP = myopathy; and BMI = body mass index.

**Table 2:** Clinical characteristics of the patients.

Variables median (min–max)	Total (n = 45)	NNP (n = 11)	NP (n = 13)	MP (n = 21)	NNP–NP p	NNP–MP p	NP–MP p
Brooke scale	1.0 (1.10–6.0)	1.0 (1.0–5.0)	1.0 (1.0–2.0)	1.0 (1.0–6.0)	0.567	0.867	0.327
Vignos scale	3.0 (1.0–10.0)	2.0 (1.0–10.0)	2.0 (1.0–9.0)	2.5 (1.0–9.0)	0.616	0.876	0.741
FIM motor score	89.0 (29.0–91.0)	85.0 (33.0–91.0)	86.0 (42.0–91.0)	90.5 (29.0–91.0)	0.723	0.655	0.424
FIM cognitive score	35.0 (18.0–35.0)	35.0 (35.0–35.0)	35.0 (30.0–35.0)	35.0 (18.0–35.0)	0.715	0.177	0.440
FIM total score	122.0 (64.0–126.0)	120.0 (68.0–126.0)	121.0 (72.0–126.0)	123.0(64.0–126.0)	0.681	0.794	0.498
BBS	47.0 (0.0–55.0)	1.0 (0.0–1.0)	50.0(47.0–53.0)	19.0 (3.0–55.0)	0.001	0.009	0.210
FAS	5.0 (0.0–5.0)	5.0 (0.0–5.0)	4.5 (1.0–5.0)	4.5 (1.0–5.0)	0.731	0.751	0.832

min = minimum; max = maximum; NNP = neuronopathy; NP = neuropathy; MP = myopathy; FIM = functional independence measurement; BBS = Berg balance scale; and FAS = functional ambulation scale.

**Table 3:** Laboratory and DEXA findings of the patients for OP assessment.

Variables median (min–max)	Total (n = 45)	NNP (n = 11)	NP (n = 13)	MP (n = 21)	NNP–NP p	NNP–MP p	NP–MP p
ALP (U/L)	73 (25–125)	68 (25–92)	71 (32–125)	81 (32–109)	0.768	0.129	0.804
Ca (mg/dL)	9.5 (7.7–10.6)	9.6 (9.08–10.6)	9.5 (8.7–9.9)	9.45 (7.70–10.6)	0.304	0.702	0.691
PTH (ng/L)	35.6 (7.8–108.6)	29.1 (15–72)	23.55 (7.8–55.2)	55.2 (18.3–108.6)	0.213	0.036	0.003
25-(OH)D (μg/L)	13.95 (3.3–48.0)	21.3 (14.6–48.0)	13.3 (4.8–40.5)	10.1 (3.30–38.0)	0.072	0.098	0.488
Lumbar spine T-score	−0.95 (−3.0/3.4)	−1 (−1.1/−0.9)	−1 (−2.7/0.6)	−0.95 (−3.0/1.4)	0.990	0.828	0.430
Hip T-score	−1.20 (−2.8/1.3)	−1.7 (−2.3/−1.1)	−0.95 (−1.8/0.7)	−1.3 (−2.80/−0.50)	0.378	0.761	0.302
ALT (U/L)	22 (7–64)	18 (9–48)	12 (10–42)	25.5 (7–64)	0.569	0.247	0.087
AST (U/L)	25 (10–88)	24 (10–61)	18 (12–36)	27.5 (15–88)	0.431	0.363	0.020

OP = osteoporosis; DEXA = dual-energy X-ray absorptiometry; min = minimum; max = maximum; NNP = neuronopathy; NP = neuropathy; MP = myopathy; ALP = alkaline phosphatase; Ca = calcium; PTH = parathyroid hormone; 25-(OH)D = 25-hydroxyvitamin-D; ALT = alanine amino-transferase; and AST= aspartate amino-transferase.

**Table 4:** Correlation between clinical and laboratory findings.

Variables	PTH r/p	25-(OH)D r/p	Lumbar spine T-score r/p	Hip T-score r/p
Age	−0.212/0.260	0.272/0.119	−0.460/0.098	−0.381/0.179
Age at diagnosis	−0.206/0.274	−0.023/0.895	−0.154/0.615	0.068/0.825
BMI	0.085/0.685	−0.176/0.361	0.163/0.596	0.504/0.066
Muscle weakness	−0.407/0.029	−0.061/0.736	−0.127/0.665	−0.025/0.931
Pain level (VAS)	−0.385/0.115	0.402/0.071	−0.006/0.986	0.082/0.822
Age independent walking ceased	0.086/0.872	0.424/0.255	0.231/0.365	0.900/0.037
Number of falls in previous 6 weeks	−0.175/0.364	0.186/0.299	−0.253/0.383	−0.604/0.022
Brooke scale	−0.224/0.243	−0.141/0.435	0.114/0.698	−0.307/0.285
Vignos scale	−0.078/0.686	−0.299/0.091	−0.015/0.960	−0.487/0.078
FIM motor score	0.384/0.043	−0.062/0.737	0.005/0.988	0.497/0.071
FIM cognitive score	−0.224/0.251	0.170/0.353	0.345/0.227	0.145/0.359
FIM total score	0.288/0.138	0.035/0.851	0.005/0.988	0.497/0.071
BBS	0.632/0.368	−0.316/0.684	0.833/0.167	0.949/0.05
FAS	0.116/0.547	0.251/0.165	−0.006/0.984	0.329/0.25

r = correlation coefficient (Pearson correlation coefficient was used for BMI and the Spearman rho coefficient was used for the other parameters); PTH = parathyroid hormone; 25-(OH)D = 25-hydroxyvitamin-D; BMI = body mass index; VAS = visual analog scale; FIM = functional independence measurement; BBS = Berg balance scale; and FAS = functional ambulation scale.
